# Validation of a binding site on the SORLA‐retromer complex that augments endosomal recycling: Implications for a novel class of AD drugs

**DOI:** 10.1002/alz.088363

**Published:** 2025-01-09

**Authors:** Kalpana M. Merchant, Olav M. Andersen, Bruce Lefker, Jane Withka, Scott A. Small, Gregory A. Petsko, Joel Klein, Rob Armstrong

**Affiliations:** ^1^ Retromer Therapeutics, New York City, NY USA; ^2^ Retromer Therapeutics, New York, NY USA; ^3^ Columbia University, New York, NY USA; ^4^ Harvard Medical School and Brigham & Women’s Hospital, Boston, MA USA

## Abstract

**Background:**

Genetic studies have established that loss of function *SORL1* gene variants are associated with Alzheimer’s disease (AD). *SORL1* encodes an endosomal trafficking receptor, SORLA, which regulates endosomal protein recycling through its interaction with the retromer core complex (consisting of VPS26, VPS35 and VPS29). Deficits in the levels and function of the SORLA‐retromer complex are thought to underlie AD. We set out to identify and validate potential small molecule binding sites on the SORLA‐retromer complex that may restore endosomal recycling and lead to disease‐modifying therapies for AD.

**Method:**

We engineered HEK293 stable cell lines expressing a *SORL1* reporter construct to monitor shedding and production of soluble SORLA (sSORLA), as indicators of endosomal recycling activity. In parallel, we synthesized cell‐permeant derivatives (R8 and TAT‐conjugations) of a retromer stabilizing cyclic peptide, RT‐L4 (Chen *et al*., Sci Adv, 2021), and its analogs, M10 and M7, that bind to the VPS26‐VPS35 interface *in vitro* at range of potencies. These peptides and several controls were tested for their ability to augment the shedding of SORLA in the cell media. Cell viability was assessed using CellTiter‐Glo and DRAQ7 staining.

**Result:**

Using a Homogenous Time‐Resolved Fluorescence (HTRF) binding assay, we confirmed that the cell‐permeant derivatives of RT‐L4, M10 and M7 bind to the retromer heterotrimer at affinities similar to the parent cyclic peptides. Treatment of HEK293 cells with cell‐permeant derivatives of these peptides, but not unmodified peptides and controls, increased sSORLA levels in the media at potencies that correlated with their binding affinity for the VPS26‐VPS35 interface. These effects were seen at concentrations that did not produce significant cell toxicity monitored by two different methods.

**Conclusion:**

These data validate the VPS26‐VPS35 interface as a target for small molecules to enhance SORLA‐retromer‐mediated recycling. The results provided a compelling rationale to conduct a high throughput screen to discover and develop a novel class of AD drugs.

